# Multi-level societies: different tasks at different social levels

**DOI:** 10.1098/rstb.2023.0274

**Published:** 2025-03-20

**Authors:** Ettore Camerlenghi, Danai Papageorgiou

**Affiliations:** ^1^ Department of Behavioural Ecology, Bielefeld University, Bielefeld 33615, Germany; ^2^ School of Biological Sciences, University of Bristol, Bristol BS8 1TQ, UK; ^3^ Department of Evolutionary Anthropology, University of Zurich, Zurich CH-8057, Switzerland

**Keywords:** multi-level sociality, partner choice, animal societies, social complexity, task performance, cooperative relationships

## Abstract

Multi-level vertebrate societies, characterized by nested social units, allow individuals to perform a wide range of tasks in cooperation with others beyond their core social unit. In these societies, individuals can selectively interact with specific partners from higher social levels to cooperatively perform distinct tasks. Alternatively, social units of the same level can merge to form higher-level associations, enabling individuals to benefit from large social units without always maintaining a large core social unit. The reasons why multi-level sociality evolves in some systems but not in others are not well understood. We propose that this is partly due to a lack of data, especially regarding the fitness consequences of cooperation at different social levels. First, we argue that in multi-level societies individual fitness benefits should increase when performing tasks in cooperation with associates from higher social levels. Second, as more multi-level societies are documented across taxa, we will continue to find similar cooperative tasks performed at each of the different social levels. By providing compelling species examples, from dolphins to fairy-wrens, we underscore that despite the diversity of multi-level social organization, convergence in task performance across social levels will become clearer as more data accumulates. Finally, we highlight the role of multi-level sociality in buffering fluctuating environmental conditions by enabling flexible social associations to emerge according to need.

This article is part of the theme issue ‘Division of labour as key driver of social evolution’.

## 1. Introduction

Multi-level sociality describes social systems that consist of groups (herein social units) that can merge in a predictable way and form distinct higher social levels, exhibiting thus fission–fusion dynamics along the boundaries of social units as per Grueter *et al*. [1]. The composition and size of social units in a multi-level social system must remain stable over time within at least two social levels [1–3], although different levels of social organization may differ in their cohesion and the stability of their membership over time [3]. The formation of distinct social levels may be driven either by active social preferences (e.g. as shaped by age, sex, genetic relatedness, or by associating with individuals with similar phenotypes) or by non-social phenomena [1,3], such as the attraction of multiple social units of the same level to the same food resources and habitat geometry [4,5]. Being composed of nested social units, multi-level societies can offer individuals the benefits of fission–fusion dynamics, such as reduced competition and increased information transmission [6]. Even when interacting at higher levels of social organization, some of the social levels remain stable and cohesive. These benefits mainly derive from having the flexibility to decide with whom to associate in response to changing social and ecological pressures [7,8], while still benefiting from strong long-term social bonds within the core social unit and weaker long-term social bonds between individuals that are members of the same higher-level unit.

Classically, the capacity to form multi-level societies was thought to be exclusive to large-brained mammals, as navigating preferential and differential dyadic relationships with conspecifics is cognitively demanding [9], both within and across levels of social organization. However, recent research on social systems of some birds and fish has provided evidence that smaller-brained species may also form multi-level societies, potentially offering valuable insights into the evolution of these societies [1,10–13]. The majority of the studies on multi-level sociality, either on large-brained mammals or beyond, have so far focused on describing the social organization [14] and the delineation of the distinct social levels (see for example [15]), rather than on the tasks that are achieved by individuals participating in them. Following Loftus *et al*. [16], we define tasks as ‘any behaviour that positively affects the fitness of conspecifics within a social group by providing a good or service to those conspecifics’. By looking beyond social organization, we expand on the idea, previously proposed but not thoroughly explored yet [2,17], that comparing across species the distinct tasks that individuals perform at different social levels can shed light on the evolution of multi-level sociality.

## 2. Different tasks at different social levels

In several eusocial insect societies, members of a social unit (e.g. a nest or colony) share a common inclusive fitness interest in their social unit achieving a specific objective (such as successfully rearing a brood cohort). Reaching this objective requires completing a series of interconnected tasks, which often leads to the emergence of division of labour between members. Splitting these component tasks between individuals can boost efficiency by allowing individuals to become specialists. Ant superorganisms, which constitute a group of individual organisms that possess the fundamental characteristics of an organism itself, as per Kennedy *et al*. [18], offer the prime example: by maximizing the colony’s reproductive output, workers increase their inclusive fitness. This implies that they maximize the sum of both their own direct and indirect fitness [18]. The ability to increase inclusive fitness through division of labour has allowed for extreme levels of behavioural and morphological specialization to evolve (see physical soldier castes in [19]). Even in social units where inclusive fitness benefits are less aligned (such as cooperatively breeding vertebrates), some degree of behavioural specialization can evolve: individuals can adopt different social roles (e.g. sentinels and babysitters in meerkats) in the course of their lifetime [20–22].

**Figure 1.**
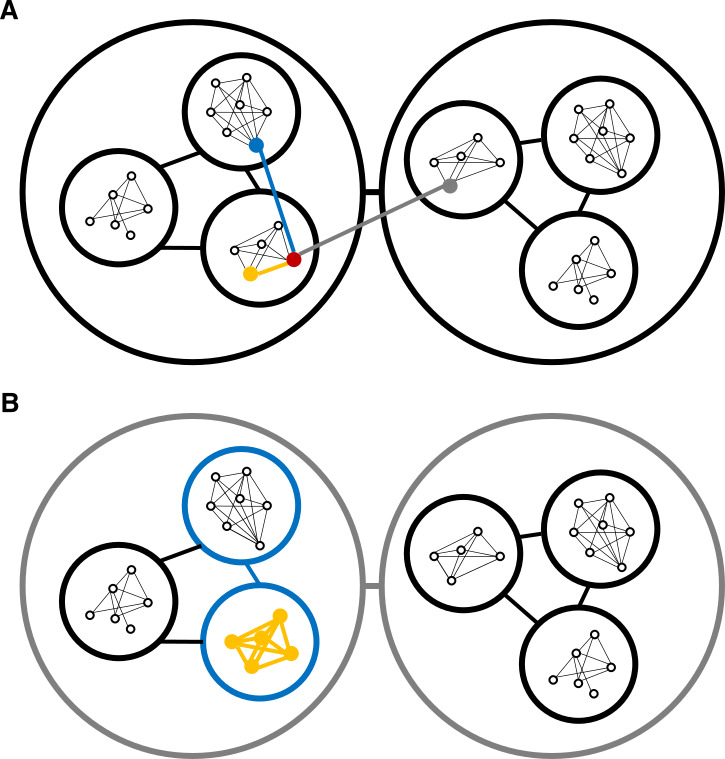
Individuals from multi-level societies perform different tasks when associating with individuals from different social levels. Here, we present one multi-level society that exhibits three levels of social associations: individuals (small white nodes with black outlines) associate preferentially with others from their own core social unit. Then, core social units (intermediate-sized nodes) associate preferentially with each other and form higher-level units (large nodes, termed as communities here), which also have contacts with other neighbouring communities. In panel (A) a focal individual (red) performs ‘Task A—yellow’ only when associating with individuals from its own core social unit, ‘Task B—blue’ when associating with individuals from its own community and ‘Task C– grey’ when associating with individuals from neighbouring communities. In panel (B) we depict a slightly different scenario where all individuals from one core social unit or community merge together with another unit of the same social level and collectively perform tasks A, B or C.

In contrast, individuals’ interests in vertebrate multi-level societies are often in conflict. For example, a helper-at-the-nest foraging to provision its siblings (usually the lowest social level beyond the pair in avian multi-level societies) may be indifferent to the success of other nests as these often contain unrelated broods. Therefore, the success of a nest may not offer any, or only limited, opportunities to increase the inclusive fitness of individuals from neighbouring nests. When labour is divided, it usually occurs within the core social unit of a multi-level society, in which there are often shared inclusive fitness benefits by completing tasks in cooperation (e.g. brood care for shared offspring or for offspring highly related to the helpers). However, despite a lack of indirect fitness benefits between individuals from different social units, cooperation across levels of social organization may regularly occur when it leads to a synergistic increase in direct fitness benefits. Multi-level sociality offers flexibility: it provides a wide and heterogeneous pool of potential partners, on different social levels, for completing specific tasks, such as obtaining information about resources or collectively defending against predation and intruders. When an individual’s interests align with those of others from this pool of potential partners, cooperative social units can form to achieve a specific objective. Cooperation between individuals from different social units (see figure 1A and bonobos [23]) or between entirely different social units (see figure 1B and dolphins [24–26]) without immediate payoff may thus be widespread in multi-level societies (see current debate on [23] and corresponding e-Letter by Connor *et al*. 2024).

In multi-level societies of vertebrates, individuals can perform a wide range of tasks in their daily routines but the tasks they perform may differ according to whom they interact with and which level of social organization they share with their co-operators (see examples in table 1). Different social levels may function as entities that collectively specialize on distinct tasks. Preferences for cooperatively completing tasks at a social level may allow individuals to gain fitness benefits that would otherwise be inaccessible. The potential benefits stemming from different levels of social organization specializing in distinct tasks suggest two predictions:

**Table 1.** Examples from vertebrates that form multi-level societies and engage in tasks with individuals in different social units. Social units are termed as is in the original studies.

		definition of social units and tasks performed within...			
**species**	**number of social levels**	...**social level 1:**	...**social level 2:**	...**social level 3:**	... **social level 4:**
hunter–gatherer societies [17,27] (*Homo sapiens*)	3 or 4	household: male–female complementarity and sex division of labour.	cluster: social units formed by extended family. Assistance from kin and intergenerational division of labour.	camp: cooperation with both related and unrelated individuals. Selection of foraging partners.	between-camp visits: information sharing and cultural innovations.
hamadryas baboons [28,29] (*Papio hamadryas*)	4	OMU: one male unit which hosts multiple females. Breeding unit.	clan: two to three OMUs led by kin males, observable during resource scarcity. Foraging as OMUs or clans.	band: multiple clans together, like troops in olive baboons. Communal sleeping, movement coordination, some affiliative interactions.	troop: two bands sharing sleeping sites. Predator detection and defence, but no other group tasks.
geladas [30] (*Theropithecus gelada*)	4	OMU: one male unit which hosts multiple females and potentially also a few follower males. Breeding unit. AMG: all-young-male units are an alternative first-level structure.	team: aggregation of two or more first-level units that associate with each other at least 90% of the time. Tasks not specified.	band: collection of first-level units that spend between 50 and 90% of their time together. Communal sleeping and foraging, not more specific tasks. Like band in hamadryas baboons.	community: the set of units with overlapping home ranges that are found together < 50% of the time. Tasks not specified.
guinea baboons [31–33] (*Papio papio*)	3	reproductive unit: one male, one to several females, young and many secondary males.	party: three to five reproductive units. Foraging, socializing, collective movement.	gang: several parties together. Predator detection and defence in communal sleeping sites.	
golden snub-nosed monkeys [34,35] (*Rhinopithecus roxellana*)	3	OMU: one male unit which hosts multiple females. Breeding unit.	band: social units formed by different OMUs. Males within bands likely defend females from bachelor males.	troop: several bands together. Females disperse between troops to breed.	
African elephants [36,37] (*Loxodonta africana*)	up to 6	breeding females with calves are listed as the first level. We kept the paper’s definition for the first level here, but we would not characterize mothers with calves as a distinct social level.	family: small size compared to third and fourth levels. Not affected by seasonality. Raising offspring and coordinating movement.	bond/kinship social units: affected by seasonality. Predator defence, territoriality, knowledge sharing and rearing of young.	clans: broader compared to third but functional differences have not been identified.
Indo-Pacific bottlenose dolphins of Shark Bay [24–26] (*Tursiops aduncus*)	3	first-order alliance: duos or trios of males consorting females in a cooperative manner.	second-order alliance: pool for forming first-order alliances. Socializing and supporting second-order allies to defend females. Stable membership.	third-order alliance: individuals from two or more second-order alliances come together and fight against rival males from neighbouring third-order alliances.	
pacific sperm whales [38,39] (*Physeter macrocephalus*)	3 to multiple	permanent social units: contain around 11 females and immatures from unrelated matrilines. Caring for offspring.	temporary intermediate social units of multiple first-level units of the same cultural clan: defence from killer whales.	clans: can contain up to 20 000 individuals. They have culturally determined vocalizations and distinct movement patterns.	there is a big difference in size from the second- to the third-level units and the literature suggests that intermediate levels might be missing.
superb fairy-wren [10,11,40] (*Malurus cyaneus*)	3	cooperatively breeding unit: individuals that assist a breeding pair to raise a brood of young.	supergroup: two neighbouring breeding units that merge stably. Tasks not specified.	community: emerges from repeated interactions between three to four breeding units and supergroups. Likely cooperative defence against predators, and communal defence against competitors.	
bell miner [41,42] (*Manorina melanophrys*)	3	cooperatively breeding unit: individuals that assist a breeding pair to raise a brood of young.	coterie or clan: occupying a discrete area within the colony. May contain one or more breeding pairs and nonbreeders. Members associate preferentially with each other, and helpers may assist more than a single pair within a coterie.	colony: a geographically discrete aggregation of between 20 and 200 individuals that together inhabit and communally defend an area against avian intruders and predators.	
vulturine guineafowl [15,43–46] (*Acryllium vulturinum*)	3	cooperatively breeding units and stable clusters of males: breeding pairs can be stable from one breeding season to the next and raise young cooperatively with specific non-parents. Clusters of males (potentially highly related) remain stable across years.	groups: can contain from 13 to 65 individuals that forage, decide collectively and move as single entities.	stable between-group associations: driven by social preferences as well as by resource abundance and distribution. They share communal roosts and information. They also form supergroups and travel together to rarely visited areas.	
cooperatively breeding cichlid fish [47–49] (*Neolamprologus pulcher*)	3	cooperatively breeding group: individuals assist a breeding pair to raise a brood of young.	colony: neighbouring breeding groups jointly defend against predators.	aggregation: feeding assemblies of members from different breeding groups exchanging social information by sporadic interactions.	

First, performing tasks in cooperation with associates from higher levels of social organization will increase fitness at the level of the individual: by exploiting the multi-level social organization, an individual can achieve tasks more efficiently than would be possible without it. For example, vulturine guineafowl (*Acryllium vulturinum*) groups (i.e. core social units) merge, thus forming a higher level of social organization, and explore largely unknown and unfamiliar areas [44]. Theory predicts that they have increased collective intelligence while navigating in the novel environment [50], but they should also be increasing their survival probabilities by being better at detecting predators [51]. In principle, this should be tested by comparing survival rates of individuals navigating novel environments only with associates from their own core social unit, as opposed to with conspecifics from additional core social units. In the cooperatively breeding cichlid fish *Neolamprologus pulcher*, which likely forms a multi-level society, breeding groups (i.e. the core social unit) exposed to a larger network of conspecifics in their colony (higher-level associations) have a higher reproductive outcome [47]. In addition, joint defence of neighbouring breeding groups can save effort of group members, leading to increased efficiency at the colony level [48]. Furthermore, it is worth examining whether lower-level social units, when isolated from the multi-level network, can complete tasks typically carried out cooperatively with individuals from other social units. Such examinations could be done with observational studies or (where feasible) in field and lab manipulations (see cichlid fish examples below). Alternatively, comparing different populations of the same species that differ in whether they form multi-level societies could allow us to identify the social and ecological conditions under which multi-level societies evolve.

Second, as multi-level societies become increasingly documented [3,10], we will consistently find that tasks are being completed cooperatively across social units at different social levels. Despite the diversity of organisms that form multi-level societies and their independent evolutionary paths, there seems to be a functional convergence in that lower levels often provide reproductive opportunities and offspring care, sometimes in the context of cooperative breeding, and higher levels facilitate processes such as information transmission, defence against competitors and predator defence [11,47,48,52]. For example, despite their phylogenetic distance, species from different taxa, such as the colonial cichlid fish *N. pulcher* [47], sperm whales (*Physeter macrocephalus*) [39], superb fairy-wrens (*Malurus cyaneus*) [11] and bell miners (*Manorina melanophrys*) [42], likely form higher-level social units to defend against predators and intruders. Additionally, in vulturine guineafowl [44] and African elephants (*Loxodonta africana*) [53], higher-level social units converge in providing individuals with the benefits of information transmission about resources, especially during harsh times. These are broad but not universal patterns, and exceptions can be found: for instance, hamadryas baboons (*Papio hamadryas*) [54], and snub-nosed monkeys (e.g. *Rhinopithecus roxellana*) [34,55] use intermediate or higher-level social units as pools for finding mating partners. Nonetheless, based on current knowledge (table 1), individuals rarely breed or care for broods with other individuals from different lower-level social units with which they form a higher-level unit, but rather join them to exchange information or mob intruders and predators [56]. Most multi-level societies have not been studied through the lenses of task performance at different levels. Therefore, evidence on the convergence of benefits provided by different levels of social organization to individuals in multi-level societies is still scarce. However, we expect that as more data accumulate, the pattern of task convergence will become clearer.

## 3. Examples from multi-level societies

Within multi-level societies, units at different social levels have been shown to perform different tasks. Individuals may selectively interact with specific individuals from other social units and engage in diverse tasks with them (figure 1A). Otherwise, entire social units interact collectively with entire other units of the same level to perform various tasks (figure 1B). These scenarios are not mutually exclusive: they are likely to depend on the type of tasks and the social level involved. We further discuss the multi-level cooperation scenarios described in figure 1 in the context of observed behaviours in wild populations of animals (such as those listed in table 1). In doing so, we hope to illustrate the breadth of the taxonomic spectrum of multi-level societies. However, this is not an exhaustive review of all known species forming multi-level societies; instead, we draw on representative examples to demonstrate that units at different social levels perform different tasks across a range of different taxa.

### (a) Indo-Pacific bottlenose dolphins

Male Indo-Pacific bottlenose dolphins (*Tursiops aduncus*) in Shark Bay of Western Australia form three alliance levels [24–26]. The first level consists of pairs or trios of males, who consort oestrus females cooperatively and get mating opportunities with them. While consorting a female, first-order allies stay very close to each other for hours to weeks. The intermediate level (second-order alliance) is the core unit of male social organization, where membership is stable across decades, but spatial cohesion is not always maintained, as second-order allies constantly split and merge with each other [26]. Second-order alliances provide a pool of individuals from which males can form first-order alliances (similar as in figure 1A). Individuals that are second-order allies socialize together and support each other when defending and stealing females from rival alliances. The third-order alliance consists of multiple second-order alliances that preferentially unite to defend females from theft attempts by rival males. Thus, in this system, males consort females together with their first-order allies and help their second- and third-order allies steal and defend females from theft by other males. In this system, cooperation occurs both within first- and second-order alliances and between third-order allies (more like the synergies in figure 1B).

### (b) Hamadryas baboons

The multi-level society of hamadryas baboons at the Awash National Park in lowland Ethiopia has four levels [28,29]. The first, known as one male unit (OMU), is the breeding unit and consists of several females and one male. Two to three OMUs led by kin males organize in ‘clans’ and forage together when resources are scarce. Multiple clans can form a ‘band’, and members from the same band coordinate movement. Multiple bands merge to form ‘troops’, the apex social level in the society. Within troops, individuals often share sleeping sites and exhibit collective predator detection and defence but no other known social tasks. In this hamadryas baboon society, in each of the four social levels, all individuals from two or more social units (e.g. OMUs) merge together, as in figure 1B.

### (c) Humans

In multi-level societies of hunter–gatherers, across several cultures, three social levels have been identified. These may correspond to three kinds of cooperative relationship: (i) male–female sex division of labour within households, (ii) assistance from kin within clusters (i.e. extended family), which form the intermediate social unit, often through intergenerational division of labour and (iii) selection of foraging partners within a camp, the upper-level social unit [17]. Finally, frequent visits between camps allow individuals to share information beyond their specific camp, creating the potential for the emergence of cultural innovations [27,57]. These distinct cooperative relationships (similar to figure 1A) might represent strategies to cope with three fundamental challenges of foraging groups in most human societies: (i) engaging in different economic activities entails differential risks and gains for women and men; (ii) obtaining resources requires a pool of diverse skills, depending on age-related individual experience, within the core social unit; and (iii) maximizing foraging effectiveness and accessing reproductive opportunities often requires forming large social units [58,59].

### (d) Superb fairy-wrens

In superb fairy-wrens, a cooperatively breeding songbird native to South-East Australia, multi-level social organization provides individuals with access to cooperative relationships that are expressed differentially across social levels [11]. At the lowest organizational level, superb fairy-wrens form breeding units that consist of a breeding pair and some helpers. During the non-breeding season (the harsher time of the year), breeding units can form both supergroups, which involve two breeding units, as well as stable higher-level social units, termed as communities [10]. These communities facilitate cooperative relationships among different breeding unit members. When breeding units entirely merge (figure 1B), they engage in defence against predators and communal defence against competitors [11]. This likely helps individuals buffer the effects of harsh environmental conditions during winter months, when individual mortality peaks [40,60].

### (e) Beyond mammal and avian societies

Although inter-group cooperation can have evolved across a range of social species, not many societies beyond mammals and birds have been discussed under the framework of multi-level sociality developed by Grueter *et al*. [1]. However, species such as the cooperatively breeding cichlid fish (*N. pulcher*) fall well within this framework. In *N. pulcher*, reproduction occurs at the lowest organizational level (the breeding unit), and predator defence involves synergies between multiple of these different breeding units. When such breeding units act together, as illustrated in figure 1B, they are termed as colonies [47]. Similarly, in the congeneric *Neolamprologus savoryi*, where groups (the higher social units) are socially and genetically structured into subgroups, members of different subgroups cooperate to defend a larger territory [61,62]. In the Australian ant *Iridomyrmex purpureus*, individuals from the same colony are spread across several separate nests, which remain socially connected [63]. Despite typically showing fidelity to a single nest within the colony, in the face of predation risk individuals across different nests within the colony cooperate to collectively defend their colony [64]. In Neotropical paper wasps (*Polistes canadensis*), workers regularly move from their home colony to neighbouring colonies, which has invited comparisons with vertebrate multi-level societies. Here, the tasks are essentially the same regardless of partners: workers perform standard worker tasks at neighbouring colonies (potentially motivated by indirect fitness benefits of helping neighbouring kin) [65]. In all the above examples, except that of Neotropical paper wasps, individuals adopt different tasks when associating with partners from different social levels. Multi-level sociality, as well as the convergence of tasks at different levels of social organization, can have evolved in different taxonomic groups that show inter-group cooperation [66]. These may include fish, eusocial insects and social shrimps. However, many animal societies still remain understudied, and often terminologies used to define social organization are taxon-specific [67]. Thus, it remains challenging to discover multi-level societies beyond the well-studied large-brained mammals, which is crucial for the development of a multi-level sociality synthesis that captures the diversity of social animals.

### (f) Emergent properties in multi-level societies

In all the above cases, synergies between individuals across different social levels in the network may facilitate the completion of qualitatively different social tasks at distinct social levels. These synergies arise from interactions among entire lower social units or specific individuals [68] and often result in collective behaviours entirely absent at the individual level. Therefore, they could be considered a form of emergent property of social aggregations. However, this does not imply that synergies and their emergent properties resulting in qualitatively different tasks in a multi-level society are inevitable features of multi-level social organization. Nonetheless, the concept of emergent properties might be an important tool for describing and exploring collective behaviour in multi-level societies, across a broad range of taxa.

## 4. Enlarging social unit size by avoiding associated costs when environmental conditions require it

Maintaining a large social unit size incurs costs and benefits that are traded off against each other, placing limits on social unit size [69–72]. Larger social units face decreased predation risk (see, for example, dilution effects [73] and the many-eyes hypothesis [51]), higher collective intelligence allowing them to solve problems [50,74,75], and are less likely to go extinct as per classic group augmentation ideas [76]. At the same time, though, large social unit size is also accompanied by larger coordination challenges [71] and intragroup competition [77]. Additionally, large social unit size poses a greater risk of infectious disease [78] and is linked to higher inequality of division of labour among members of the same social unit [79]. Despite these costs, some animal societies invest resources to accommodate excess social unit members, potentially necessary under certain circumstances, as discussed in [80,81], following the concept of redundancy.

In contrast, multi-level societies, characterized by preferential fission–fusion dynamics among social units, facilitate flexible adjustments in social unit size in response to changing environmental conditions, social competition and resource availability. For instance, in hamadryas baboons [28] and African elephants [37], intermediate social units may fragment into smaller units, mitigating competition during periods of resource scarcity. Conversely, in some species, core social units may coalesce during times of scarcity. This collective behaviour, observed in species such as vulturine guineafowl [44] and killer whales (*Orcinus orca*) [82], facilitates the performance of specific tasks as the need arises, offering, for example, safety against predators and enhancing information transmission regarding the location of vital resources. Territorial species that form multi-level societies, on the other hand, may exhibit increased intergroup tolerance during harsh conditions with limited resources, enabling individuals to exploit larger areas when resources within their territory are scarce [10]. In summary, within the context of multi-level societies, individuals within a social unit can derive benefits like those of larger social units by forming preferential associations with specific individuals from other social units of the same or higher levels (figure 1A), or by cooperating with entire other social units when necessary to perform distinct tasks (figure 1B).

## 5. Outstanding questions

Much remains unclear about why and how multi-level societies have evolved, under what conditions there is convergence of tasks at different levels of social organization across species and to what extent cooperating with individuals from different levels of social organization increases individual fitness. Recent studies have shown that there is scope for detailed observation of multi-level interactions across a wider taxonomic breadth, extending beyond mammals [10,15,48]. Additionally, field manipulations could quantify the decision-making and fitness consequences of social connections at different levels, but to achieve this, studies on multi-level sociality should move beyond characterizing multi-level social organization and rather explore the tasks performed at different levels. Finally, evolutionary modelling could shed light on the transition to a multi-level social organization [66].

### (a) Questions regarding different tasks at different levels

— How common are multi-level societies and are they all characterized by different tasks at different levels? In which cases are there no differentiation between levels in the tasks they perform?
— Are there fitness consequences of losing access to different levels of a multi-level society (see [25])?
— Is the early evolution of multi-level sociality driven by individuals choosing different partners for different tasks? Or do individuals tactically choose their partners only once they find themselves in a multi-level society that has largely arisen for other (passive) reasons? If the former, which tasks and which partnerships were decisive?
— Has division of labour evolved among different units of the same organizational level, where, for example, one first-level social unit specializes in one task and another first-level social unit in another task, in any multi-level societies beyond humans?
— Does coordinated collective action among members of separate social units in a multi-level society qualify as genuine polyadic cooperation or do individuals simply show independent yet simultaneous defensive action in response to intruders or threats (see [48])?

### (b) Broader open questions on multi-level sociality

— Are the evolutionary trajectories to multi-level societies similar across taxa or are they idiosyncratic to each case?
— To what extent is life in an incipient multi-level society an adaptive choice or a burden for different individuals?
— How do power asymmetries shape multi-level societies? Does multi-level sociality increase the scope for achieving private aims only for those with the social power to make or break social connections across levels?
— Are multi-level societies more resilient to environmental shocks compared to unilevel societies?
— Why is there interspecific variation in whether harsh environmental conditions drive social units to split or merge?
— Do individuals in structurally complex multi-level societies have knowledge of the social structure they are embedded within and is such knowledge useful across taxa that form multi-level societies?

## 6. Conclusions

Of the presented examples and case studies, many support the idea that multi-level societies, through cooperative relationships across diverse social levels, enable individuals to accomplish distinct social tasks [1]. By providing individuals within a social unit access to a familiar pool of potential social partners beyond their core social unit, these societies efficiently address challenges of group living, such as safety against predators, competitors and information sharing, without the cost of maintaining a large, potentially sub-optimal and stable social unit size. As a promising avenue for further exploration (see §5), we encourage the empirical investigation of two broad hypotheses: (i) task performance in cooperation with associates from higher levels of social organization increases individual fitness, and (ii) as additional multi-level societies are documented across taxa, we expect to identify similar cooperative tasks being performed at each of the distinct social levels. By systematically mapping the individual benefits associated with different cooperative relationships across social levels in various taxa, we can gain crucial insights into the social complexity of multi-level societies and into what drives their emergence across the animal kingdom.

## Data Availability

This article has no additional data.
